# Identification of 3-Oxindole Derivatives as Small Molecule HIV-1 Inhibitors Targeting Tat-Mediated Viral Transcription

**DOI:** 10.3390/molecules27154921

**Published:** 2022-08-02

**Authors:** Dong-Eun Kim, Young Hyun Shin, Jung-Eun Cho, Subeen Myung, Hong Gi Kim, Kyung-Chang Kim, Chul Min Park, Cheol-Hee Yoon

**Affiliations:** 1 Division of Chronic Viral Diseases, Center for Emerging Virus Research, Korea National Institute of Health, 187 Osongsaengmyeong 2-ro, Cheongju 363951, Korea; dongeunkim@korea.kr (D.-E.K.); yhshin80@korea.kr (Y.H.S.); joytodeath@korea.kr (K.-C.K.); 2 Center for Convergent Research of Emerging Virus Infection (CEVI), Korea Research Institute of Chemical Technology, 141 Gajeong-ro, Daejeon 34114, Korea; jungeun@krict.re.kr (J.-E.C.); msb25@krict.re.kr (S.M.); tenork@krict.re.kr (H.G.K.); 3 Medicinal Chemistry and Pharmacology, Korea University of Science and Technology, Daejeon 34114, Korea

**Keywords:** human immunodeficiency virus type 1 (HIV-1), 3-oxindole, antiviral activity, HIV-1 transcription

## Abstract

The heterocyclic indole structure has been shown to be one of the most promising scaffolds, offering various medicinal advantages from its wide range of biological activity. Nonetheless, the significance of 3-oxindole has been less known. In this study, a series of novel 3-oxindole-2-carboxylates were synthesized and their antiviral activity against human immunodeficiency virus-1 (HIV-1) infection was evaluated. Among these, methyl (*E*)-2-(3-chloroallyl)-4,6-dimethyl-one (**6f**) exhibited the most potent inhibitory effect on HIV-1 infection, with a half-maximal inhibitory concentration (IC_50_) of 0.4578 μM but without severe cytotoxicity (selectivity index (SI) = 111.37). The inhibitory effect of these compounds on HIV-1 infection was concordant with their inhibitory effect on the viral replication cycle. Mode-of-action studies have shown that these prominent derivatives specifically inhibited the Tat-mediated viral transcription on the HIV-1 LTR promoter instead of reverse transcription or integration. Overall, our findings indicate that 3-oxindole derivatives could be useful as a potent scaffold for the development of a new class of anti-HIV-1 agents.

## 1. Introduction

Acquired immune deficiency syndrome (AIDS) is a major global problem threatening public health. The condition is caused by human immunodeficiency virus type-1 (HIV-1) infection [1]. Currently, six classes of drugs have been developed to treat individuals infected with HIV-1, and a combination of at least two classes of drugs, called combination anti-retroviral therapy (cART), are used to suppress viral replication efficiently [2]. Nonetheless, these treatments allow persistent HIV-1 infection and are frequently inadequate with regard to the emergence and transmission of drug-resistant mutations [3,4,5]. Therefore, global efforts to develop new classes of drugs that inhibit the HIV-1 life cycle are ongoing in medicinal research.

Heterocyclic chemical structures, acting as highly functionalized scaffolds, have been widely adopted in medicinal and pharmaceutical research. In this context, 2-oxindole (also referred to as 2-indolinone or oxindole) comprises a six-membered benzene ring fused to a five-membered nitrogen-containing pyrrole ring connecting a carbonyl group at C2 position. The structure is found widely in plants and mammals where it plays an important role in the maintenance of physiological homeostasis. Several derivatives of 2-oxindole obtained from natural sources are used in traditional medicinal practices, with biological activities including anticancer, anti-inflammation, antipyretic, and antimicrobial action [6,7,8]. Based on their diverse biological activities, several novel 2-oxindole compounds have been recently developed as marketed drugs, such as nintedanib, indolidan, ropinirole, and ziprasidone [9]. Furthermore, a series of 2-oxindole derivatives were synthesized and evaluated for antiviral activity against HIV-1, with inhibitory effects on HIV-1 reverse-transcriptase (RTase) [10]. A natural compound bearing 3-oxindole that is closely related to 2-oxindole was initially identified as a new class of potent antitumor agent including duocarmycin A and duocamycin SA [11]. Several 3-oxindole-2-carboxylates were newly synthesized and exhibited potent antimicrobial activity against bacterial infection of plants [12] or usability as a sensitive fluorescent probe to detect fat deposits in an animal model [13]. In spite of several potent activities of 3-oxindole-2-carboxylates, their antiviral effect has not been reported before. In this study, we synthesized a series of novel derivatives of 3-oxindole and screened for active derivatives against HIV-1 infection. In addition, we found that the mode of action underlying anti-HIV-1 activity of the 3-oxindole derivatives was associated with inhibition of Tat-mediated viral transcription.

## 2. Results and Discussion

### 2.1. Chemistry

**Scheme 1:**
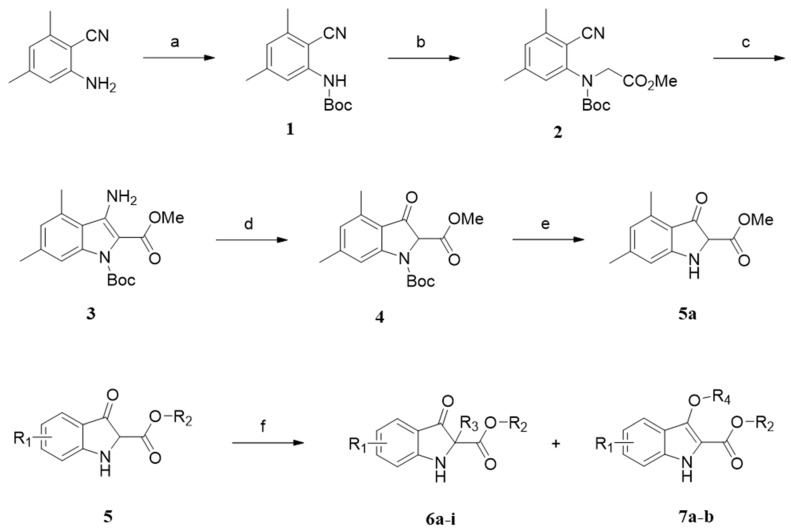
General synthesis of alkyl 3-oxindole-2-carboxylates used in the present study. Reagents and conditions: (**a**) Boc_2_O, DMAP, TEA, acetonitrile, 60 °C; (**b**) Methyl bromoacetate, Cs_2_CO_3_, DMF, room temperature; (**c**) LiHMDS, THF, −78 °C to room temperature; (**d**) Dowex™-50, MeOH, water, room temperature; (**e**) trifluoroacetic acid, dichloromethane, room temperature; (**f**) Alkyl halides, K_2_CO_3_, acetone.

The synthetic routes used for compounds **6a**–**i** and **7a**–**b** are depicted in Scheme 1. A representative intermediate 3-oxindole-2-carboxylate (**5a**) was prepared from 2-amino-4,6-dimethylbenzonitrile, which was protected via reaction with Boc_2_O in the presence of 4-dimethylaminopyridine (DMAP) and triethylamine (TEA) to provide compound **1**. Alkylation of **1** with methyl bromoacetate in the presence of Cs_2_CO_3_ led to compound **2**. Dieckmann condensation of **2** by treatment of LiHMDS provided 3-amino-4,6-dimethylindoline-2-carboxylate **3**. Treatment of **3** with Dowex™ 50 in aqueous methanol led to 3-oxo-indole-2-carboxylate **4**, which was deprotected with trifluoroacetic acid (TFA) to give key intermediate **5a**. 3-oxindole-2-carboxylates **5** were alkylated in the presence of K_2_CO_3_ to provide O- or C-alkylated derivatives **6a**–**i** and **7a**–**b** (see Table 1).

### 2.2. Evaluation of Anti-HIV-1 Activity

**Figure 1:**
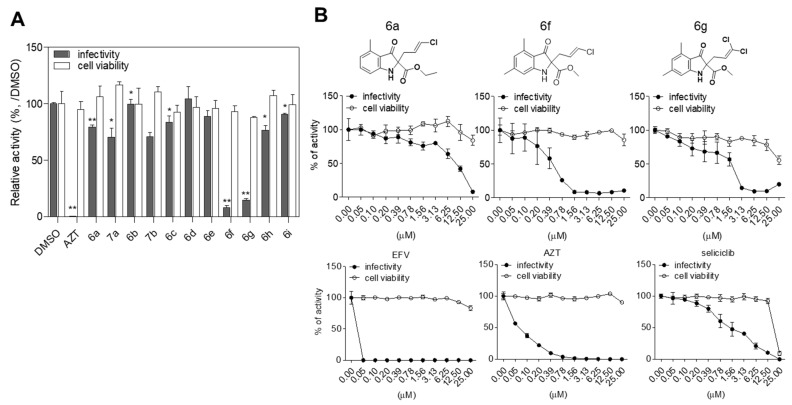
Comparison of the anti-HIV-1 effect of 3-oxindole and 3-oxy-indole derivatives (**A**,**B**) TZM-bl cells (1 × 10^4^ cells/well) cultured in 96-well plate were treated with 3 μM (**A**) or two-fold serial dilutions * *p* < 0.05, ** *p* < 0.01 (**B**) of indicated compound, and then infected with the HIV-1_NL4-3_ strain at an MOI of 1. After 48 h, viral infectivity was determined with a firefly luciferase assay kit. Cell viability was assessed using a resazurin-based cell viability reagent in parallel with infectivity assay. EFV, AZT and seliciclib were used as the experimental control, and the data are presented as the mean ± SD (*n* = 3).

To explore whether the synthetic 3-oxindole-2-carboxylates are capable of inhibiting HIV-1 infection, all 11 3-oxindole derivatives were used to treat TZM-bl cells with infection of HIV-1_NL4-3_ at a final concentration of 3 μM, and the inhibitory effect on HIV-1 infectivity was compared with that of azidothymidine (AZT), a marketed inhibitor of HIV-1 infection, as a control. Among the tested substances, compound **6f**, which has a 3-chloroallyl group at the 2-position and 4,6-dimethyl substituents of 3-oxindole-2-carboxylates, exhibited a nearly complete inhibitory effect on HIV-1 infectivity without cytotoxicity. The compound **6g**, which has a 3,3-dichloroallyl group at the 2-position, also showed a notable inhibitory effect on HIV-1 infection. Additionally, compound bearing 4-methyl (**6a**) with the 3-chloroallyl group at the 2-position and compounds that have the 3-chloroallyl group at the 3-position of 3-oxodindoline-2-carboxylates (**7a** and **7b**) showed a slight inhibitory effect of approximately 20% (Figure 1A and Table 1).

To determine the potency of the 3-oxindole-2-carboxylates on anti-HIV-1 activity, we analyzed their dose response with 1:2 serially diluted compounds at an initial concentration of 25 μM, which is relevant to medicinal application. The most significant activity, as shown in Table 2, was demonstrated by compound **6f**, with a half-maximal inhibitory concentration (IC_50_) value of 0.4578 μM. The compound **6g** also showed potent activity with an IC_50_ value of 1.276 μM and higher toxicity compared with **6f**. The compound **6a** exhibited a moderate inhibitory effect on HIV-1 infection, with an IC_50_ value of 13.685 μM (Figure 1B and Table 2). Of the compounds that showed slight activity (Figure 1A), **7a** and **7b** gradually inhibited HIV-1 infectivity in a dose-dependent manner up to a concentration of 25 μM. While these inhibition ratios reached almost 50%, **6h** did not show further increased inhibitory effect even at concentrations higher than 1.56 μM (Supplementary Materials Figure S1A).

The CC_50_ (concentration that reduces cell viability by 50%) value of these compounds could not be determined at the concentration tested because of the low toxicity of these compounds (Table 2, and Figure 1B). Thus, the CC_50_ values of the most promising compounds (**6a**, **6f**, and **6g**) were determined through serial dilution of the compounds starting from 100 μM. Based on the determined CC_50_ values, the selectivity indices (SIs, the ratio of IC_50_ to CC_50_) of compounds **6a**, **6f**, and **6g** were determined as 6.38, 111.37, and 34.19, respectively. As controls, the IC_50_ values of efavirenz (EFV) and AZT were determined to be <0.05 μM and 0.079 μM, and SIs were >683.86 and >1265.5, respectively, similar to that reported in previous studies (Table 2) [14,15]. The other compounds showed no effect on either viral infectivity or cytotoxicity (*data not* *shown*). These data demonstrate that the 3-oxindole scaffold has potent intrinsic activity against HIV-1 infection. The compounds showing prominent activity have several similar structural features. All have the 3-chloroallyl group at the 2-position of the 3-oxindole; the compounds substituted with propyl (**6i**) or allyl (**6h**) at the 2-position exhibited decreased anti-HIV-1 activity. Moreover, 4,6-dimethyl substitution on 3-oxindole-2-carboxylate (**6f** and **6g**) might be critical for anti-HIV-1 activity because either no substitution (**6d**) or monosubstitution with a methoxy (**6b**) or methyl (**6e**) at C5 exhibited reduced activities.

**Figure 2:**
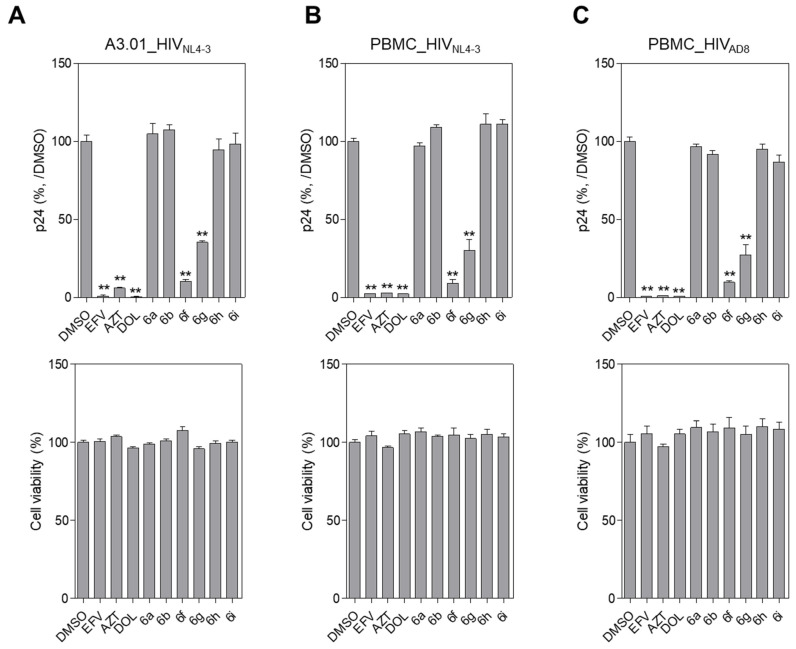
Inhibitory effect on viral replication (**A**,**B**) A3.01 cells (**A**) and PBMCs (**B**) were infected with HIV-1_NL4-3_ at an MOI of 0.1 and then treated with compounds (1.5 μM) for 72 h. The inhibitory effect of the compounds on viral replication was determined by measuring the amount of p24 using an HIV-1 p24 ALPHALISA™ kit. Cell viability was determined as described in Materials and Methods. (**C**) PBMCs infected with HIV-1_AD8_ at an MOI of 0.1 were treated with indicated compounds (1.5 μM) for 72 h, and the p24 levels and cell viability were determined as shown above. Data are expressed as a value relative to the vehicle (DMSO)-treated controls, as the mean ± SD (*n* = 3). ** *p* < 0.01 compared with the cells treated with the vehicle.

To verify the inhibitory effect of prominent 3-oxindole-2-carboxylates on the overall HIV-1 life cycle, HIV-1 replication assays were performed with A3.01 T cell lines (Figure 2A) and peripheral blood mononuclear cells (PBMCs) (Figure 2B) infected with CXCR4 tropic HIV-1_NL4-3_ in the presence of the test compounds. The compound **6f** exhibited near-complete abrogation of viral replication at a concentration of 1.5 μM, **6g** exhibited half inhibition of viral replication and the other compounds (**6a**, **6b**, **6h**, and **6i**) had no inhibition of viral replication at the concentration tested without cytotoxicity, all of which was consistent with the inhibitory effects of the compounds on viral infectivity. Moreover, prominent compounds **6f** and **6g** inhibited replication of the CCR5 tropic HIV-1_Ad8_ strain in the PBMCs similar to that shown in the CXCR4 tropic HIV-1_NL4-3_ strain at the same concentration (Figure 2C).

**Figure 3:**
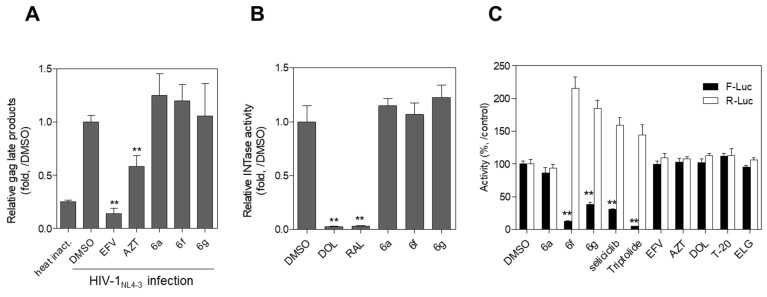
Determination of mode of action of the 3-oxindole derivatives (**A**) A3.01 cells treated with 10 μM of the indicated compounds were infected with HIV-1_NL4-3_. At 16 h after infection, the cells were harvested, and the levels of HIV-1 RT product were determined as described in Materials and Methods. (**B**) An INTase assay was performed with 10 μM of the indicated compounds according to the manufacturer’s protocol. (**C**) bl-DTR cells were treated with 3 μM of the indicated compounds and then cultured in the presence of doxycycline (50 ng/mL). At 24 h after treatment, firefly luciferase (F-Luc) and renilla luciferase (R-Luc) activities were determined using the Dual-Glo™ luciferase assay system. The data (**A**–**C**) are presented as a value relative level to the vehicle (DMSO)-treated controls, as the mean ± SD (*n* = 3). ** *p* < 0.01 compared with the cells treated with vehicle. T20: a fusion inhibitor, ELG: elvitegravir.

To further address the mode of action linked to anti-HIV-1 activity of prominent 3-oxindole derivatives, a HIV-1 reverse transcriptase (RTase) assay and an integrase assay were performed initially. RTase inhibitors (EFV and AZT) and integrase inhibitors (dolutegravir: DOL and raltegravir: RAL) inhibited the activities of RTase and integrase, respectively, but our compounds did not inhibit either of the two HIV-1 enzymatic activities (Figure 3A,B).

**Figure 4:**
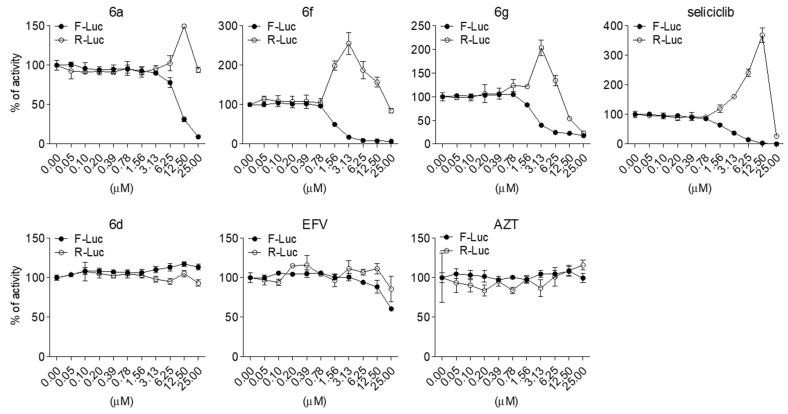
Concentration–response of the 3-oxindole derivatives on the inhibition of Tat-mediated HIV-1 transcription. bl-DTR cells treated with two-fold serial dilutions of each compound were cultured in the presence of doxycycline (final concentration, 50 ng/mL). At 24 h after treatment, the activities of firefly luciferase (F-Luc, closed circle) and renilla luciferase (R-Luc, open circle) were determined using the Dual-Glo™ luciferase assay system. Seliciclib was used as an experimental positive control, and the data are presented as the mean ± SD (*n* = 3).

To examine whether the compounds inhibit HIV-1 transcription induced by the viral transcriptional factor Tat, a Tat-mediated transcription assay was performed in bl-DTR cells, which express simultaneously Tat-induced F-Luc and R-Luc upon doxycycline treatment (see 3. Materials and Methods), in the presence of our compounds. As shown in Figure 3C, Tat-induced transcriptional activity (F-Luc) was significantly reduced by treatment with our lead compounds without a decrease in R-Luc activity. Seliciclib and triptolide also exhibited specific inhibition of Tat-induced HIV-1 transcription as controls (Figure 3C). Thus, we conducted a concentration response assay of the prominent derivatives to evaluate their activity against Tat-mediated HIV-1 transcription. Notably, compound **6f** inhibited the Tat-induced transcriptional activity completely at a concentration of approximately 6 μM, with an IC_50_ value of 1.2 μM. In fact, the inhibitory effect was stronger than that shown by well-known Tat inhibitor seliciclib (IC_50_ value of 2.41 μM). The application of compounds **6a** and **6g** resulted in IC_50_ values of 9.53 μM and 2.85 μM, respectively (Figure 4 and Table 3). Compounds that showed no inhibitory effect on HIV-1 infectivity did not inhibit Tat-induced HIV-1 transcription, as exemplified by **6d**. Their Tat inhibitory effects closely corresponded to their activities against HIV-1 infection. These data revealed that derivatives bearing a 3-oxindole backbone inhibited Tat-induced viral transcription, which led to their inhibitory effects on HIV-1 infection. As shown in Figure 4, the increase in R-Luc activity, in phase with a decrease in F-Luc activity, might have been because of an excess of common factor(s) for transcription caused by the suppression of F-Luc expression as described previously [16,17], while the decrease in R-Luc activity in high dosages (12.5 μM and 25 μM) of compounds **6g** and seliciclib might be relevant to cellular toxicity and/or off-target effects as described in the report in developing the cellular system (Figure 1B) [18].

Current HIV-1 drugs target the specific viral replication steps of entry, reverse transcription, integration, and protease-mediated viral maturation. However, these antiretroviral treatments often lead to the emergence of drug-resistant mutations and adverse effects, as well as reservoirs of cells infected with latent HIV-1 that are not eradicated by current treatments [19]. Therefore, efforts are needed to develop new HIV-1 drugs to control the spread of HIV/AIDS.

In this study, a series of compounds containing a heterocyclic 3-oxindole core were synthesized and screened for inhibition of HIV-1 infection. The 3-oxindole core is structurally similar to indoles, which have occupied a salient place in medicinal chemistry because of their diverse biological activities as to anticancer, antimicrobial, antioxidation, and anti-HIV-1 effects. In particular, extensive investigations on the indole scaffold has led to the development of potent RTase inhibitors including derivatives of EFV, AZT, and nevirapine (NVP) [20,21], inhibitors of HIV-1 attachment to host membrane as exemplified by the BMS-663068 [22], integrase strand-transfer inhibitors (INSTIs) such as fluorobenzylindole derivatives [23], and inhibitors of HIV-1 transcription such as aristolactam [17] and 18BIOder [24]. Furthermore, 2-oxindole derivatives were also suggested to act as inhibitors of RTase [25]. In spite of the structural similarity of 3-oxindole to indole/ 2-oxindole, the potency of 3-oxindole in the developmental field of anti-HIV-1 agents has not been explored. In this study, we demonstrate for the first time that the 3-oxindole scaffold can provide an ability to inhibit HIV-1 infection. Although the anti-HIV-1 activity of 3-oxindole-2-carboxylates was facilitated by substituting 3-chloroallyl and 3,3-dichloroallyl at the 2-position with 4,6-dimethyl substituents (**6f** and **6g**, respectively), the SAR study using more diverse substitutions on 3-oxindole-carboxylates may be further required to improve their potency against HIV-1 infection and reduce the toxicity. The mode of action of the lead compounds against HIV-1 infection has been revealed to inhibit the Tat-mediated viral transcription rather than RTase inhibition shown in several indole/2-oxindole derivatives against HIV-1. In addition, we conducted several biological experiments to determine whether these compounds inhibit Tat activity directly. However, these prominent compounds (**6f** and **6g**) did not inhibit the cellular expression level of Tat protein, the nuclear localization of Tat, the interaction between Tat and cyclin T1 which is an essential component of super elongation complex (SEC) to facilitate viral transcription, and the association of Tat with TAR RNA, which is a step critical for the SEC to access viral promoter LTR. These results might indicate that these 3-oxindole derivatives do exert for Tat inhibition indirectly (*manuscript in preparation*) [26]. Details of the mechanism based on inhibition of Tat-mediated transcription remain to be elucidated in a further study. Less active compounds (**7a** and **7b**) exhibited gradual inhibition of HIV-1 infectivity in a dose-dependent manner but do not affect the Tat-mediated viral transcription (Supplementary Materials Figure S1B). These data suggest that the 3-oxindole scaffold may have multifaceted modes of action to inhibit HIV-1 infection.

## 3. Materials and Methods

### 3.1. General Chemical Information

All reagents used were of the highest grade commercially available and used as purchased from Aldrich, TCI, Alfa Aesar, or Combi block. Thin-layer chromatography was carried out using Merck Kiesel gel 60 F_254_ plates. Chromatographic purification was carried out using silica gel (Merck, 70–230 mesh). The purity of the synthesized compounds was ascertained by proton nuclear magnetic resonance (^1^H NMR) and ^13^C NMR spectroscopy, measured with a Bruker Avance 300-MHz spectrometer with tetramethylsilane as an internal standard. Chemical shifts in the NMR data are given as a chemical shift (δ) in ppm, multiplicity (if applicable), coupling constants (*J*) in Hz (if applicable), and integration (if applicable). Multiplicities are indicated by s (singlet), d (doublet), t (triplet), q (quartet), and m (multiplet). MS spectra were collected with a Waters’ LC-MS system (ZMD-1) and used to confirm ≥95% purity of each compound. The column used was an L-column 2 ODS (3.0 × 50 mm I.D., CERI, Saitama, Japan) with a temperature of 40 °C and a flow rate of 1.2 mL/min. Mobile phase A was 0.05% TFA in ultrapure water, and mobile phase B was 0.05% TFA in acetonitrile, which was increased linearly from 5% to 90% over 2 min and was 90% over the next 1.5 min, after which the column was equilibrated to 5% for 0.5 min. High-resolution mass spectroscopy (HRMS) was carried out at Takeda Analytical Laboratories, Ltd., without optimizing the yields.

#### 3.1.1. *tert*-Butyl (2-cyano-3,5-dimethylphenyl) carbamate (**1**)

The 2-Amino-4,6-dimethylbenzonitrile (3.2 g, 22 mmol) in anhydrous acetonitrile (30 mL) was treated with di-*tert*-butyl dicarbonate (6.54 g, 30 mmol), DMAP (2.44 g, 20 mmol), and trimethylamine (5 mL, 41 mmol). The resulting reaction mixture was stirred at 60 °C for 24 h under a flow of nitrogen. The mixture was extracted with ethyl acetate (30 mL), 1N HCl and washed with distilled water (10 mL) and brine (10 mL). The organic layer was dried on MgSO_4_, filtered, and the residue was purified by silica gel flash column chromatography (hexane to EtOAc/ hexane = 1:2). Compound **1** (2.1 g, 40%) was isolated pure as a yellow solid; mp 156–157 °C; *R_f_* = 0.3 (EtOAc/ hexane = 1:7).

^1^H NMR (400 MHz, DMSO) δ 9.24 (s, 1H), 7.12 (s, 1H), 7.04 (s, 1H), 2.41 (s, 3H), 2.31 (s, 3H), 1.47 (s, 9H). ^13^C NMR (126 MHz, DMSO) δ 153.04, 143.53, 126.92, 122.83, 79.77, 28.02, 21.22, 20.17. HRMS (ESI): *m*/*z* calculated for C_15_H_18_N_2_O_2_ [M]^+^ 246.1368, found 246.1358.

#### 3.1.2. Methyl-*N*-(*tert*-butoxycarbonyl)-*N*-(2-cyano-3,5-dimethylphenyl)-glycinate (**2**)

A solution of compound **1** (750 mg, 3 mmol) in DMF (10 mL) was treated with methyl bromoacetate (0.56 mL, 6 mmol) and cesium carbonate (1.2 g, 4 mmol) at 25 °C under N_2_ and the mixture was stirred for 4 h. To the mixture was added H_2_O (20 mL), and the organic layer was extracted with ethyl acetate (20 mL) and washed with brine (20 mL). The resulting organic layer was dried on MgSO_4_, filtered, and the residue was purified by silica gel flash column chromatography (hexane to EtOAc/ hexane = 1:5). Compound **2** (850 mg, 90%) was isolated as a yellow solid; mp 160 °C; *R_f_* = 0.5 (EtOAc/ hexane = 1:2).

^1^H NMR (400 MHz, DMSO) δ 7.18 (q, *J* = 7.6, 6.6 Hz, 2H), 4.35 (d, *J* = 14.5 Hz, 2H), 3.68 (s, 2H), 2.44 (s, 3H), 2.34 (s, 3H), 1.42 (s, 3H), 1.34 (s, 6H). ^13^C NMR (126 MHz, DMSO) δ 169.78, 152.79, 144.05 (d, *J* = 15.3 Hz), 142.09, 129.22, 125.09, 116.28, 109.52, 81.19 (d, *J* = 10.9 Hz), 59.81, 52.05 (d, *J* = 11.6 Hz), 51.03, 27.53, 21.20, 20.81.

HRMS (ESI): *m*/*z* calculated for C_17_H_22_N_2_O_4_ [M]^+^ 318.1580, found 246.1588.

#### 3.1.3. 1-(*tert*-Butyl) 2-methyl 4,6-dimethyl-3-oxindole-1,2-dicarboxylate (**4**)

Compound **2** (1.9 g, 6 mmol) was dissolved in anhydrous THF (10 mL) and freshly prepared lithium bis(trimethylsilyl)amide solution (LiHMDS) (1 M in THF, 7 mL, 7 mmol) in anhydrous THF (10 mL) under N_2_ was added at 0 °C. The reaction mixture was stirred for 24 h at 25 °C before the reaction was quenched with the addition of saturated aqueous NH_4_Cl (10 mL). The mixture was extracted with ethyl acetate (20 mL) and washed with distilled water (20 mL) and brine (20 mL). The organic layer was dried on MgSO_4_, filtered, and then concentrated *in vacuo* to provide crude 1-(*tert*-butyl) 2-methyl 3-amino-4,6-dimethylindoline-1,2-dicarboxylate **3** (1.88 g), which was used directly in the next step.

A solution of compound **3** (1.88 g, 6 mmol) in CH_3_OH (20 mL) and H_2_O (10 mL) at 25 °C was treated with Dowex™ 50 (**6g**), and the reaction mixture was stirred for 24 h. The mixture was diluted with ethyl acetate (20 mL) and filtered to remove the resin. The filtrate was washed with brine (20 mL), dried with MgSO_4_, and concentrated in vacuo. The mixture was also purified by silica gel flash column chromatography (hexane to EtOAc/ hexane = 1:5) to provide compound **4** (420 mg, 22%) as a yellow solid; mp 180 °C; *R_f_* = 0.2 (EtOAc/ hexane = 1:5).

^1^H NMR (400 MHz, DMSO) δ 7.87 (s, 1H), 6.86 (s, 1H), 5.14 (s, 1H), 3.74 (s, 3H), 2.47 (s, 3H), 2.39 (s, 3H), 1.44 (s, 9H). ^13^C NMR (126 MHz, DMSO) δ 206.63, 189.67, 126.24, 113.34, 82.22, 52.96, 30.75, 27.61, 22.14, 17.76.

HRMS (ESI): *m*/*z* calculated for C_17_H_21_NO_5_ [M]^+^ 319.1420, found 319.1415.

#### 3.1.4. Methyl 4,6-dimethyl-3-oxindole-2-carboxylate (**5a**)

A solution of compound **4** (400 mg, 1.3 mmol) in CH_2_Cl_2_ (10 mL) was treated with trifluoroacetic acid (1.4 mL) at 0 °C under N_2_, and the reaction mixture was stirred at room temperature for 24 h. NaOH (2 M) solution was added until the pH reached up to 11. The mixture was extracted with ethyl acetate (10 mL), distilled water (10 mL), and brine (10 mL). The organic layer was dried on MgSO_4_, filtered, and the residue was purified by silica gel flash column chromatography (hexane to EtOAc/hexane = 1:5). The compound **5a** (100 mg, 35%) was isolated pure as a yellow solid; *R_f_* = 0.2 (EtOAc/hexane = 1:5).

^1^H NMR (400 MHz, DMSO) δ 10.70 (s, 1H), 8.58 (s, 1H), 6.86 (s, 1H), 6.53 (s, 1H), 3.84 (s, 3H), 2.56 (s, 3H), 2.31 (s, 3H). ^13^C NMR (126 MHz, DMSO) δ 162.60, 145.69, 136.14, 135.76, 131.63, 121.67, 114.93, 109.37, 107.84, 51.00, 21.53, 18.70. MS (ESI+): *m*/*z* 220.2 [M+H]^+^.

#### 3.1.5. Ethyl (E)-2-(3-chloroallyl)-4-methyl-3-oxindole-2-carboxylate (6a) and ethyl (e)-3-((3-chloroallyl)oxy)-4-methyl-1h-indole-2-carboxylate (**7a**)

To ethyl 3-hydroxy-4-methyl-1*H*-indole-2-carboxylate (250 mg, 1.1 mmol) in acetone (10 mL) was added *trans*-1,3-dichloropropene (0.21 mL, 1.1 mmol) and K_2_CO_3_ (950 mg, 1.1 mmol), and the resulting reaction mixture was stirred at 60 °C for 24 h under a flow of N_2_. The mixture was extracted with ethyl acetate (20 mL), distilled water (20 mL), and brine (20 mL). The organic layer was dried on MgSO_4,_ and after filtration, the solvent was removed to give a residue. The reaction mixture was purified by preparative HPLC (Shim-pack PREP-ODS, H_2_O/CH_3_CN/CH_3_OH = 40:30:30 to H_2_O/CH_3_CN/CH_3_OH = 1:49.5:49.5, flow rate = 12 mL/min, 40 °C, λ = 254 nm, retention time: 30 min) to provide compound **6a** (125 mg, 46%) and **7a** (86 mg, 26%) as brown solids.

Compound **6a**: mp >300 °C; *R_f_* = 0.2 (EtOAc/ hexane = 1:5). ^1^H NMR (400 MHz, DMSO) δ (**6a**) 7.78 (s, 1H), 7.38–7.29 (m, 1H), 6.76 (d, *J* = 8.2 Hz, 1H), 6.52 (d, *J* = 7.2 Hz, 1H), 6.41–6.32 (m, 1H), 5.80–5.68 (m, 2H), 4.18–4.02 (m, 2H), 2.84 (m, 1H), 2.40 (s, 3H), 1.13 (t, *J* = 7.1 Hz, 3H). ^13^C NMR (126 MHz, DMSO) δ 160.11, 141.15, 132.35, 131.22, 128.80, 126.92, 121.98, 120.63, 118.75, 114.12, 113.31, 73.48, 31.14, 18.24, 15.23.

HRMS (ESI): *m*/*z* calculated for C_15_H_16_ClNO_3_ [M+H]^+^ 294.0897, found 294.1064.

Compound **7a**: mp 226 °C; *R_f_* = 0.2 (EtOAc/ hexane = 1:5). ^1^H NMR (400 MHz, DMSO) δ (**7a**) 11.35 (s, 1H), 7.19 (d, *J* = 8.3 Hz, 1H), 7.12 (dd, *J* = 8.4, 6.8 Hz, 1H), 6.78 (d, *J* = 6.8 Hz, 1H), 6.71–6.62 (m, 1H), 6.28 (dt, *J* = 13.1, 6.5 Hz, 1H), 4.61 (dd, *J* = 6.6, 1.4 Hz, 2H), 4.34 (q, *J* = 7.1 Hz, 2H), 2.59 (s, 3H), 1.35 (t, *J* = 7.1 Hz, 3H). ^13^C NMR (126 MHz, DMSO) δ 160.51, 143.35, 134.39, 131.02, 129.98, 125.52, 121.66, 120.67, 119.45, 115.62, 110.31, 73.48, 60.21, 18.54, 14.33.

HRMS (ESI): *m*/*z* calculated for C_15_H_16_ClNO_3_ [M+H]^+^ 294.0897, found 294.0874.

#### 3.1.6. Methyl (E)-2-(3-chloroallyl)-5-methoxy-3-oxindole-2-carboxylate (**6b**) and methyl (e)-3-((3-chloroallyl)oxy)-5-methoxy-1h-indole-2-carboxylate (**7b**)

To methyl 3-hydroxy-5-methyl-1*H*-indole-2-carboxylate (250 mg, 1.1 mmol) in acetone (10 mL) was added *trans*-1,3-dichloropropene (0.21 mL, 1.1 mmol) and K_2_CO_3_ (950 mg, 1.1 mmol) and the resulting reaction mixture was stirred at 60 °C for 24 h under a flow of N_2_. The mixture was extracted with ethyl acetate (20 mL), distilled water (20 mL), and brine (20 mL). The organic layer was dried on MgSO_4_ and filtered. The residue was purified by preparative HPLC (Shim-pack PREP-ODS, H_2_O/CH_3_CN/CH_3_OH = 40:30:30 to H_2_O/CH_3_CN/CH_3_OH = 1:49.5:49.5, flow rate = 12 mL/min, 40 °C, λ = 254 nm, retention time: 30 min) to afford compound **6b** (163 mg, 50%) and **7b** (100 mg, 30%) as brown solids.

Compound **6b**: mp > 300 °C; *R_f_* = 0.25 (EtOAc/ hexane = 1:5). ^1^H NMR (400 MHz, DMSO) δ (**6b**) 7.47 (s, 1H), 6.97 (d, *J* = 8.9 Hz, 1H), 6.90 (d, *J* = 2.7 Hz, 1H), 6.35 (d, *J* = 13.1 Hz, 1H), 5.70 (dt, *J* = 13.2, 7.6 Hz, 1H), 3.72 (s, 3H), 3.62 (s, 3H), 2.92–2.78 (m, 2H), 2.74 (s, 1H). ^13^C NMR (126 MHz, DMSO-*d*_6_) δ 195.69, 168.23, 158.32, 152.62, 128.40, 127.24, 120.81, 118.12, 114.27, 104.31, 73.13, 55.51, 52.88, 35.15.

HRMS (ESI): *m*/*z* calculated for C_14_H_14_ClNO_4_ [M]^+^ 295.0611, found 295.0632.

Compound **7b**: mp > 300 °C; *R_f_* = 0.2 (EtOAc/ hexane = 1:5). ^1^H NMR (400 MHz, DMSO) δ (**7b**) 11.35 (s, 1H), 7.27 (d, *J* = 8.9 Hz, 1H), 7.01 (d, *J* = 2.4 Hz, 1H), 6.96–6.89 (m, 1H), 6.62 (d, *J* = 13.4 Hz, 1H), 6.29 (dt, *J* = 13.2, 6.6 Hz, 1H), 4.70–4.65 (m, 2H), 3.86 (s, 3H), 3.78 (s, 3H). ^13^C NMR (126 MHz, DMSO) δ 160.21, 141.33, 133.31, 131.12, 128.98, 120.63, 119.46, 118.41, 115.32, 109.52, 70.48, 58.21, 30.2.

HRMS (ESI): *m*/*z* calculated for C_14_H_14_ClNO_4_ [M]^+^ 295.0611, found 295.0614.

#### 3.1.7. Methyl 5-methoxy-3-oxo-2-propylindoline-2-carboxylate (**6c**)

To a solution of methyl 3-hydroxy-5-methyl-1*H*-indole-2-carboxylate (50 mg, 0.23 mmol) in acetone (10 mL) was added iodopropane (0.04 mL, 0.41 mmol) and potassium carbonate (0.19 g, 1.37 mmol) at room temperature, and the resulting mixture was stirred at 60 °C for 8 h. The mixture was diluted with ethyl acetate and washed with water. The organic layer was dried on MgSO_4_ and filtered. The residue was purified by preparative HPLC (Shim-pack PREP-ODS, H_2_O/CH_3_CN/CH_3_OH = 40:30:30 to H_2_O/CH_3_CN/CH_3_OH = 1:49.5:49.5, flow rate = 12 mL/min, 40 °C, λ = 254 nm, retention time: 30 min) to provide compound **6c** (27 mg, 45%) as a yellow solid; mp 117–118 °C; *R_f_* = 0.3 (EtOAc/ hexane = 1:7). ^1^H NMR (400 MHz, DMSO) δ 7.18 (s, 1H), 7.01 (s, 1H), 6.89 (d, *J* = 2.6 Hz, 2H), 3.72 (d, *J* = 5.0 Hz, 3H), 3.59 (d, *J* = 11.0 Hz, 3H), 2.14 (s, 2H), 1.31 (s, 2H), 0.87 (s, 3H). ^13^C NMR (126 MHz, DMSO-*d*_6_) δ 197.60, 167.13, 158.22, 150.44, 127.10, 126.24, 119.51, 119.08, 114.68, 102.34, 75.01, 54.51, 51.28, 32.25.

HRMS (ESI): *m*/*z* calculated for C_14_H_17_NO_4_ [M]^+^ 263.1158, found 263.1930.

#### 3.1.8. Methyl (E)-2-(3-chloroallyl)-5-methyl-3-oxindole-2-carboxylate (**6d**)

To methyl 3-hydroxy-5-methyl-1*H*-indole-2-carboxylate (600 mg, 3 mmol) in acetone (10 mL) was added *trans*-1,3-dichloropropene (0.55 mL, 3 mmol) and K_2_CO_3_ (2.49 g, 3 mmol). The resulting reaction mixture was stirred at 60 °C for 24 h under a flow of N_2_, then diluted with ethyl acetate and washed with water. The organic layer was dried on MgSO_4_ and then filtered. The residue was purified by preparative HPLC (Shim-pack PREP-ODS, H_2_O/CH_3_CN/CH_3_OH = 40:30:30 to H_2_O/CH_3_CN/CH_3_OH = 1:49.5:49.5, flow rate = 12 mL/min, 40 °C, λ = 254 nm, retention time: 30 min) to provide compound **6d** (325 mg, 39%) as a brown solid; mp 106–107 °C; *R_f_* = 0.3 (EtOAc/ hexane = 1:4). ^1^H NMR (400 MHz, DMSO) δ 11.25 (s, 1H), 7.41 (s, 1H), 7.27 (s, 1H), 7.08 (s, 1H), 6.60 (s, 1H), 6.27 (s, 1H), 4.70 (s, 2H), 3.85 (s, 2H), 2.37 (s, 3H). ^13^C NMR (126 MHz, DMSO) δ 161.02, 141.78, 132.83, 130.20, 128.42, 127.56, 121.71, 120.27, 118.43, 114.98, 112.59, 72.11, 51.49, 21.12.

HRMS (ESI): *m*/*z* calculated for C_13_H_12_ClNO_3_ [M]^+^ 265.0506, found 265.0612.

#### 3.1.9. Methyl (E)-2-(3-chloroallyl)-3-oxindole-2-carboxylate (**6e**)

To methyl 3-hydroxy-1*H*-indole-2-carboxylate (480 mg, 2.5 mmol) in acetone (10 mL) was added *trans*-1,3-dichloropropene (0.46 mL, 2.5 mmol) and K_2_CO_3_ (2.07 g, 2.5 mmol). The resulting reaction mixture was stirred at 70 °C for 24 h under a flow of N_2_, then diluted with ethyl acetate and washed with water. The organic layer was dried on MgSO_4_ and filtered. The residue was purified by preparative HPLC (Shim-pack PREP-ODS, H_2_O/CH_3_CN/CH_3_OH = 40:30:30 to H_2_O/CH_3_CN/CH_3_OH = 1:49.5:49.5, flow rate = 12 mL/min, 40 °C, λ = 254 nm, retention time: 30 min) to provide compound **6e** (275 mg, 50%) as a brown solid. mp > 300 °C; *R_f_* = 0.25 (EtOAc/ hexane = 1:4). ^1^H NMR (400 MHz, DMSO) δ 11.39 (s, 1H), 7.63 (s, 1H), 7.38 (s, 1H), 7.24 (s, 1H), 7.07 (s, 1H), 6.63 (s, 1H), 6.27 (s, 1H), 4.72 (dd, *J* = 6.6, 1.4 Hz, 2H), 3.87 (s, 3H). ^13^C NMR (101 MHz, DMSO) δ 161.42, 142.65, 134.69, 130.58, 125.95, 122.22, 120.63, 119.91, 115.38, 113.23, 72.63, 51.97. 30.1.

HRMS (ESI): *m*/*z* calculated for C_14_H_14_ClNO_3_ [M]^+^ 279.0662, found 279.0734.

#### 3.1.10. Methyl (E)-2-(3-chloroallyl)-4,6-dimethyl-3-oxindole-2-carboxylate (**6f**)

To methyl 4,6-dimethyl-3-oxindole-2-carboxylate (**5a**) (480 mg, 2.5 mmol) in acetone (10 mL) was added *trans*-1,3-dichloropropene (0.085 mL, 0.9 mmol) and K_2_CO_3_ (0.38 g, 2.75 mmol). The resulting reaction mixture was stirred at 60 °C for 24 h under a flow of N_2_, then diluted with ethyl acetate and washed with water. The organic layer was dried on MgSO_4_ and then filtered. The residue was purified by preparative HPLC (Shim-pack PREP-ODS, H_2_O/CH_3_CN/CH_3_OH = 40:30:30 to H_2_O/CH_3_CN/CH_3_OH = 1:49.5:49.5, flow rate = 12 mL/min, 40 °C, λ = 254 nm, retention time: 30 min) to provide compound **6f** (32 mg, 24%) as a yellow solid. mp > 300 °C; *R_f_* = 0.2 (EtOAc/ hexane = 1:5). ^1^H NMR (400 MHz, DMSO-*d*_6_) δ 7.69 (s, 1H), 6.56 (s, 1H), 6.38–6.31 (m, 2H), 5.70 (dt, *J* = 13.1, 7.6 Hz, 1H), 3.61 (s, 3H), 2.81 (ddd, *J* = 14.2, 7.5, 1.5 Hz, 1H), 2.30 (d, *J* = 41.8 Hz, 7H). ^13^C NMR (126 MHz, DMSO-*d*_6_) δ 195.03, 168.32, 163.18, 148.56, 138.57, 127.38, 121.32, 120.70, 114.02, 109.66, 72.44, 52.81, 35.37, 21.83, 17.58.

HRMS (ESI): *m*/*z* calculated for C_15_H_16_ClNO_3_ [M]^+^ 293.0819, found 293.0825.

#### 3.1.11. Methyl 2-(3,3-dichloroallyl)-4,6-dimethyl-3-oxindole-2-carboxylate (**6g**)

To methyl 4,6-dimethyl-3-oxindole-2-carboxylate (**5a**) (50 mg, 0.25 mmol) in acetone (10 mL) was added 1,1,3-trichloropropene (0.05 mL, 0.45 mmol) and K_2_CO_3_ (0.19 g, 1.37 mmol). The resulting reaction mixture was stirred at 60 °C for 24 h under a flow of N_2_, then diluted with ethyl acetate and washed with water. The organic layer was dried on MgSO_4_ and filtered. The residue was purified by preparative HPLC (Shim-pack PREP-ODS, H_2_O/CH_3_CN/CH_3_OH = 40:30:30 to H_2_O/CH_3_CN/CH_3_OH = 1:49.5:49.5, flow rate = 12 mL/min, 40 °C, λ = 254 nm, retention time: 30 min) to provide compound **6g** (5 mg, 7%) as a yellow solid; mp > 300 °C; *R_f_* = 0.2 (EtOAc/ hexane = 1:5). ^1^H NMR (400 MHz, DMSO-*d*_6_) δ 7.76 (d, *J* = 2.3 Hz, 1H), 6.58 (s, 1H), 6.39 (s, 1H), 5.87 (td, *J* = 7.1, 2.1 Hz, 1H), 3.62 (d, *J* = 2.1 Hz, 3H), 2.90 (ddd, *J* = 15.3, 7.1, 2.1 Hz, 1H), 2.66–2.60 (m, 1H), 2.36 (d, *J* = 2.2 Hz, 3H), 2.26 (d, *J* = 2.2 Hz, 3H). ^13^C NMR (126 MHz, DMSO-*d*_6_) δ 168.18, 124.82, 121.56, 120.98, 109.71, 71.76, 52.94, 34.57, 21.84, 17.60.

HRMS (ESI): *m*/*z* calculated for C_15_H_15_Cl_2_NO_3_ [M]^+^ 327.0429, found 327.0430.

#### 3.1.12. Methyl 2-allyl-4,6-dimethyl-3-oxindole-2-carboxylate (**6h**)

To methyl 4,6-dimethyl-3-oxindole-2-carboxylate (**5a**) (50 mg, 0.25 mmol) in acetone (10 mL) was added allyl bromide (0.04 mL, 0.46 mmol) and K_2_CO_3_ (0.19 g, 1.37 mmol). The resulting reaction mixture was stirred at 60 °C for 24 h under a flow of N_2_, then diluted with ethyl acetate and washed with water. The organic layer was dried on MgSO_4_ and then filtered. The residue was purified by preparative HPLC (Shim-pack PREP-ODS, H_2_O/CH_3_CN/CH_3_OH = 40:30:30 to H_2_O/CH_3_CN/CH_3_OH = 1:49.5:49.5, flow rate = 12 mL/min, 40 °C, λ = 254 nm, retention time: 30 min) to provide compound **6h** (46 mg, 50%) as a yellow solid; mp 147 °C; *R_f_* = 0.2 (EtOAc/ hexane = 1:7). ^1^H NMR (400 MHz, DMSO-*d*_6_) δ 7.65 (s, 1H), 6.54 (s, 1H), 6.34 (s, 1H), 5.55 (ddt, *J* = 17.2, 10.2, 7.1 Hz, 1H), 5.16–5.09 (m, 1H), 5.02 (dd, *J* = 10.2, 2.1 Hz, 1H), 3.61 (s, 3H), 2.77 (dd, *J* = 13.9, 7.0 Hz, 1H), 2.54 (d, *J* = 7.3 Hz, 1H), 2.35 (s, 3H), 2.24 (s, 3H). ^13^C NMR (126 MHz, DMSO-*d*_6_) δ 195.84, 169.09, 163.60, 148.75, 138.87, 132.08, 121.50, 119.88, 114.67, 109.95, 73.22, 53.11, 39.02, 22.25, 18.01.

HRMS (ESI): *m*/*z* calculated for C_15_H_17_NO_3_ [M]^+^ 259.1208, found 259.1233.

#### 3.1.13. Methyl 4,6-dimethyl-3-oxo-2-propylindoline-2-carboxylate (**6i**)

To methyl 4,6-dimethyl-3-oxindole-2-carboxylate (**5a**) (50 mg, 0.25 mmol) in acetone (10 mL) was added iodopropane (0.04 mL, 0.41 mmol) and K_2_CO_3_ (0.19 g, 1.37 mmol). The resulting reaction mixture was stirred at 60 °C for 24 h under a flow of N_2_, then diluted with ethyl acetate and washed with water. The organic layer was dried on MgSO_4_ and filtered. The residue was purified by preparative HPLC (Shim-pack PREP-ODS, H_2_O/CH_3_CN/CH_3_OH = 40:30:30 to H_2_O/CH_3_CN/CH_3_OH = 1:49.5:49.5, flow rate = 12 mL/min, 40 °C, λ = 254 nm, retention time: 30 min) to provide compound **6i** (30 mg, 50%) as a yellow solid; mp 132–133 °C; *R_f_* = 0.2 (EtOAc/ hexane = 1:7). ^1^H NMR (400 MHz, DMSO-*d*_6_) δ 7.63 (s, 1H), 6.53 (s, 1H), 6.33 (s, 1H), 3.60 (s, 3H), 2.35 (s, 3H), 2.24 (s, 3H), 1.99–1.91 (m, 1H), 1.77–1.69 (m, 1H), 1.16 (ddd, *J* = 25.9, 12.9, 6.4 Hz, 2H), 0.83 (t, *J* = 7.3 Hz, 3H). ^13^C NMR (126 MHz, DMSO-*d*_6_) δ 195.99, 169.05, 163.25, 148.20, 138.41, 120.93, 114.28, 109.43, 73.43, 52.58, 36.62, 21.81, 17.58, 16.56, 13.98.

HRMS (ESI): *m*/*z* calculated for C_15_H_19_NO_3_ [M]^+^ 261.1365, found 261.1369.

### 3.2. Biological Assays

#### 3.2.1. Cells, Virus, and Reagents

TZM-bl and bl-DTR cells were cultured in Dulbecco’s modified Eagle’s medium supplemented with 1% penicillin–streptomycin and 10% (*v*/*v*) heat-inactivated fetal bovine serum (all obtained from Gibco-BRL, Gaithersburg, MD, USA). The bl-DTR cells were additionally supplemented with 1 μg/mL puromycin and 200 μg/mL zeocin. The PBMCs were purchased from AllCells (Alameda, CA, USA) and cultured, as described previously [16]. HIV-1 clones NL4-3 and AD8, as well as TZM-bl and A3.01 cells, were obtained from the National Institute of Health’s AIDS Research and Reference Reagent Program (NIH, Bethesda, MD, USA). Seliciclib (roscovitine, CYC202), AZT, EFV, RAL, and DOL were purchased from Sigma Aldrich (St. Louis, MO, USA).

#### 3.2.2. Assay for HIV-1 Infectivity

TZM-bl cells (also referred as JC53BL-13) expressing human CD4, CXCR4, and CCR5 in order to facilitate HIV-1 infection, which contain the *firefly luciferase* (F-Luc) gene under the control of HIV-1 long terminal repeat (LTR) promoter was used to determine inhibitory effect of the compounds on HIV-1 infection, as described previously with minor modifications [27,28]. In brief, 1 × 10^4^ cells were cultured in 96-well plates for 24 h and then treated with compounds at a final concentration of 3 μM (Figure 1A) or with 1:2 serially diluted compounds at concentrations ranging from 0 to 25 μM (dose-response assay; Figure 1B and Table 2). Without replacement of a culture medium, the cells were then infected with the HIV-1_NL4-3_ strain at a multiplicity of infection (MOI) of 1 to determine the exact inhibitory effect of compounds on a single round infection. After 48 h, the inhibitory effect of the compounds was determined using a Bright Glo luciferase assay kit (Promega). The infectivity data are presented as a percentage relative to the DMSO control (vehicle). The inhibitory effects of the compounds on viral replication were determined with some minor modifications, as described previously [17,29]. In brief, 5 × 10^4^ cells/ well of A3.01 cells were infected with HIV-1_NL4-3_ at an MOI of 0.1 with the spinoculation at 300 *g* for 2 h at 25 °C. PBMCs were pre-activated with phytohemagglutinin M (PHA-M) for 3 days. The pre-activated 4 × 10^5^ of PBMCs were washed with fresh media to remove excess PHA-M and then infected with HIV-1_NL4-3_ or HIV-1_AD8_ as that of A3.01 infection. After infection, the infectious media were replaced with fresh media (supplemented with international 10 units (IU)/mL of IL-2 for PBMCs containing 1.5 μM of compounds. At 72 h after infection, the inhibitory effect of the compounds on viral replication was determined by measuring the amount of p24, an HIV capsid protein, using an HIV-1 p24 ALPHALISA™ kit (PerkinElmer, Waltham, MA, USA). Cell viability was determined by using the resazurin-based PrestoBlue™ cell viability reagent (Thermo Fisher Scientific, Waltham, MA, USA) according to the manufacturer’s protocols.

#### 3.2.3. Assay for Tat-Mediated HIV-1 Transcription

bl-DTR (TZM-bl-derived dual Tat reporter) cells derived from TZM-bl cells by insertion of two doxycycline-inducible lentiviral vector-expressing flag-tagged tat and Renilla-luciferase (R-Luc) genes were used to determine simultaneously both activities of Tat-mediated HIV-1 transcription and general cellular transcription, as described previously [18]. Determination of the inhibitory effect of the compounds on Tat-mediated transcription was performed as described previously. In brief, 1 × 10^4^ of bl-DTR cells cultured in 96-well plates were treated with compounds at a final concentration of 3 μM (Figure 3C) or with 1:2 serially diluted compounds at concentrations ranging from 0 to 25 μM (Figure 4 and Table 3). Subsequently, doxycycline was treated at a final concentration of 50 ng/mL to induce Tat-mediated F-Luc and R-Luc. At 24 h after treatment, the activities of F-Luc and R-Luc were determined by using a Dual-Glo™ luciferase assay system (Promega, Madison, WI, USA) according to the manufacturer’s protocols. The data are presented as a percentage relative to the DMSO control (vehicle) in the presence of doxycycline, and the experiment was performed in triplicate.

#### 3.2.4. Assay for HIV-1 Reverse Transcription (RT) Activity

Activity of HIV-1 reverse transcription was determined, as described previously [17], with minor modifications. In brief, after treatment with DNase I, 2 × 10^6^ of A3.01 cells were treated with the indicated compounds prior to infection with HIV-1 NL4-3 (at an MOI ratio of 1). Sixteen hours after infection, cytosolic DNA from the cells was isolated and the levels of reverse transcription (RT) products were determined by a quantitative PCR (qPCR). The primers for RT products were 5′-GGTCCAAAATGCGAACCCAG (forward) and 5′-TCTTGCTTTATGGCCGGGTC (reverse). To determine the relative levels of the RT products, rRNA from the lysed cells were analyzed with one-step quantitative RT-PCR using the following primer sets for 18S rRNA: 5′-GTAACCCGTTGAACCCCATT (forward) and 5′-CCATCCAATCGGTAGTAGGG (reverse). The relative level of each RT product was analyzed by using the delta-delta CT method, as described previously.

#### 3.2.5. Assay for HIV-1 Integrase Activity

The integrase activity was determined using an HIV-1 integrase assay kit (XpressBio, Frederick, MD, USA), according to the manufacturer’s protocols. In brief, a 96-well plate coated with donor substrate (DS) DNA was blocked with blocking buffer for 30 min at 37 °C. One hundred μL of HIV-1 integrase diluted with 1:300 onto reaction buffer was added to the plate, and the plate was then incubated for 30 min at 37 °C. Subsequently, 50 μL of compounds were added to the plate at final concentrations of 10 and 20 μM. At 5 min after addition of compounds, 50 μL of target substrate DNA diluted at 1:100 was added to the plate and incubated for 30 min at 37 °C. The plate was washed and then incubated with 100 μL of horse radish peroxidase antibody solution for 30 min at 37 °C. Integrase activity was assessed by measuring the peroxidase activity reacted with 3,3′,5,5′-tetramethylbenzidine substrate at optical density 450 nM. The experiment was performed in triplicate.

#### 3.2.6. Statistical Analysis

All data are expressed as the mean ± SD (*n* = 3), and the data were compared using a student’s *t*-test, with * *p* < 0.05, ** *p* < 0.01 considered as being statistically significant. All statistical analyses were performed using the Prism software (v.5.0; GraphPad Software, San Diego, CA, USA).

## 4. Conclusions

A series of 3-oxindole-carboxylates were synthesized and their anti-HIV-1 activities were tested using TZM-bl cells with infection of HIV-1. The compound **6f** showed the most prominent inhibitory effect on HIV-1 infection (IC_50_ = 0.4578 μM and SI = 111.37), and **6a** and **6g** were moderately active (**6a**; IC_50_ = 13.685 μM and SI = 6.38, **6g**; IC_50_ = 1.276 μM and SI = 34.19). Anti-HIV-1 activity of these compounds was validated based on the viral replication in CD4+ T cell lines and primary PBMCs infected with either X4 tropic or R5 tropic HIV-1. Anti-HIV-1 activity of these compounds was closely associated with the specific inhibition of Tat-mediated viral transcription. Together, our results demonstrate that some derivatives bearing the 3-oxindole core can be regarded as promising compounds for the development of novel anti-HIV-1 agents.

## Figures and Tables

**Table 1:** Alkylation of 3-oxindole-2-carboxylates (5) and inhibitory effect on HIV-1 infectivity.

Alkyl 3-Oxindole-2-Carboxylates (5)	Product		Yield (%)	Inhibition of HIV-1 Infectivity (%)	Cell Viability (%)
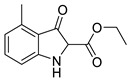 **5a**	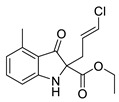	**6a**	46%	20.42 ± 1.69	106.26 ± 9.33
	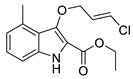	**7a**	26%	29.58 ± 8.08	116.76 ± 2.70
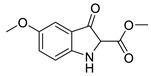 **5b**	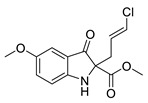	**6b**	50%	0.55 ± 4.34	99.64 ± 14.19
	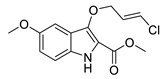	**7b**	30%	28.97 ± 3.78	110.47 ± 4.96
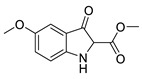 **5c**	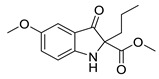	**6c**	45%	16.20 ± 5.52	92.70 ± 5.77
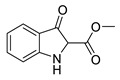 **5d**	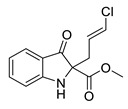	**6d**	39%	0.00 ± 10.66	96.96 ± 9.15
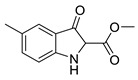 **5e**	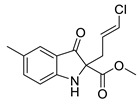	**6e**	50%	11.49 ± 5.66	95.66 ± 7.32
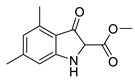 **5f**	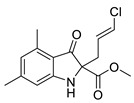	**6f**	24%	91.87 ± 1.78	92.95 ± 5.39
	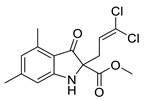	**6g**	7%	85.39 ± 1.44	87.90 ± 0.82
	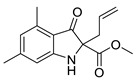	**6h**	50%	23.56 ± 4.46	107.13 ± 4.73
	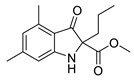	**6i**	50%	9.15 ± 0.96	99.37 ± 8.83
	Azidothymidine (AZT)			99.65 ± 0.048	94.75 ± 7.21

The effects of compounds (including AZT) on HIV-1 infection and cell viability were determined in TZM-bl cells treated at a concentration of 3 μM with infection of HIV-1 _NL4-3_ at an MOI of 1.

**Table 2:** Concentration–responses of 3-oxindole-2-carboxylates on cytotoxicity and anti-HIV-1 activity.

Compound	IC_50_(μM) ^a^	CC_50_(μM) ^b^	SI ^c^
**6a**	13.685 ± 2.5526	87.3833 ± 12.6685	6.38
**6f**	0.4578 ± 0.0227	50.99 ± 4.4357	111.37
**6g**	1.276 ± 0.9589	43.6357 ± 10.5195	34.19
EFV	<0.05	34.1933 ± 0.59	>683.86
AZT	0.079 ± 0.0237	>100	>1265.5

The inhibitory effects on inhibition of HIV-1 infection and cell viability were determined in TZM-bl cells with infection of HIV-1 _NL4-3_ at an MOI of 1. ^a^ IC_50_: half-maximal inhibitory concentration. ^b^ CC_50_: concentration that reduces cell viability by 50%. ^c^ SI: selectivity index, the ratio of IC_50_ to CC_50_.

**Table 3:** Concentration–responses of 3-oxindole-2-carboxylates on the inhibition of Tat-mediated HIV-1 transcription.

Compound	IC_50_ (μM) ^a^
**6a**	9.53 ± 0.48
**6f**	1.20 ± 0.04
**6g**	2.85 ± 0.63
seliciclib	2.41 ± 0.07

The compounds were assessed in bl-DTR cells using a concentration–response test. ^a^ IC_50_: half-maximal inhibitory concentration.

## Data Availability

The data presented in this study are available on request from the corresponding author.

## Supplementary Materials

The following supporting information can be downloaded at: https://www.mdpi.com/article/10.3390/molecules27154921/s1, Figure S1: Concentration–response of the 3-oxindole derivatives.
