# Supplementary material for: Identification of 3-Oxindole Derivatives as Small Molecule HIV-1 Inhibitors Targeting Tat-Mediated Viral Transcription
Source: Molecules. 2022 Aug 2;27(15):4921. doi: 10.3390/molecules27154921 (PMC9370035; doi:10.3390/molecules27154921)
Supplement: Supplementary file 1 [file molecules-27-04921-s001.zip › molecules-1828269-supplementary.pdf]

Supplementary Figure

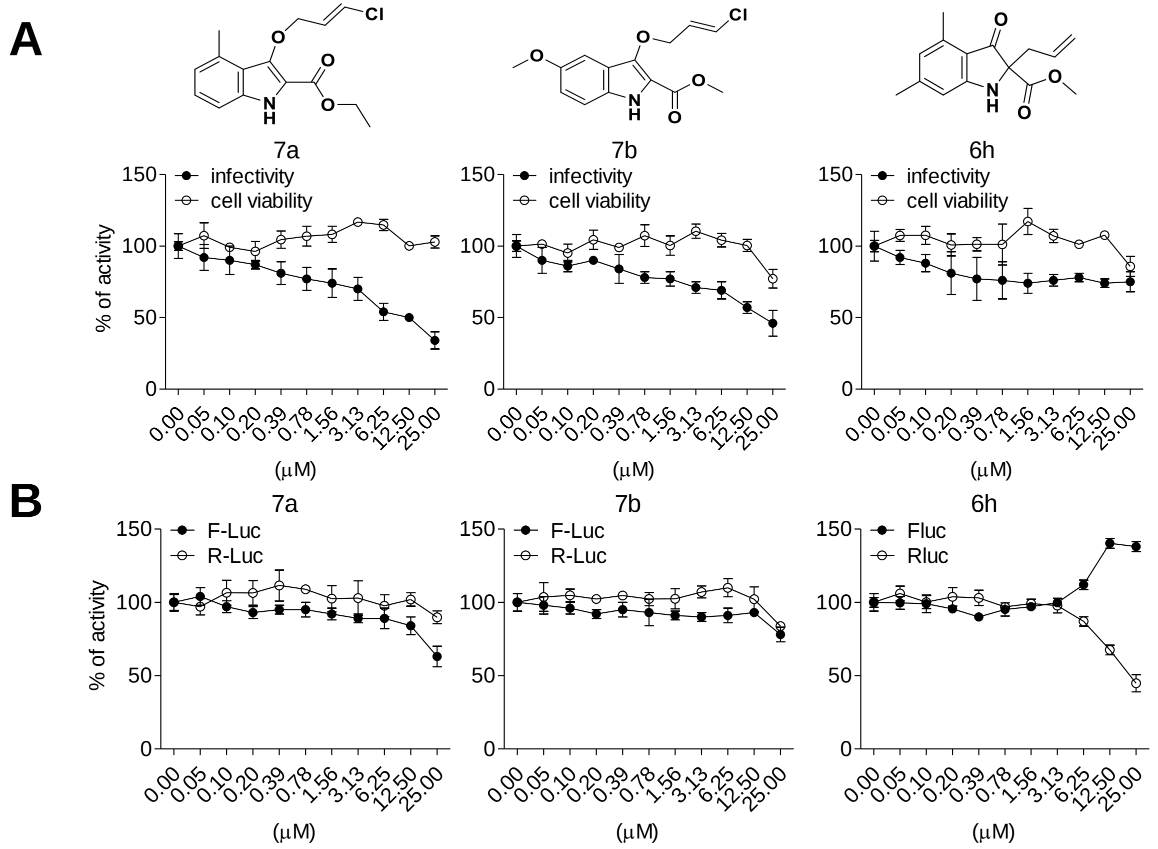

Figure S1. Concentration–response of the 3-oxindole derivatives

(A) TZM-bl cells (1×10^4) cultured in 96 well plate were treated with two-fold serial dilutions of indicated compound prior to infection with the HIV-1_{NL4-3} strain at a MOI of 1. After 48 h, viral infectivity was determined with a firefly luciferase assay kit. Cell viability was assessed using Prestoblue in parallel with infectivity assay. The data are presented as the mean ± SD (n = 3). **(B)** bl-DTR cells (1×10^4) treated with two-fold serial dilutions of each compound were cultured in the presence of doxycycline (final concentration, 50 ng/mL). At 24 h after treatment, the activity of firefly luciferase (F-Luc, closed circle) and renilla luciferase (R-Luc, open circle) was determined using the Dual-Glo Luciferase assay system. The data are presented as the mean ± SD (n = 3).
